# Management of a unique case of talon cusp, dens invaginatus associated with other dental anomalies

**DOI:** 10.11604/pamj.2022.41.326.34689

**Published:** 2022-04-22

**Authors:** Devendra Ishwarlal Nagpal, Ayushi Shashikant Gurharikar

**Affiliations:** 1 Department of Paediatric and Preventive Dentistry, Vidya Shikshan Prasarak Mandals´ Dental College and Research Centre, Nagpur, Maharashtra, India

**Keywords:** Dens invaginatus, talons cups, hypoplastic mass, composite buildup, pediatric dentistry

## Image in medicine

An 11-year-old girl reported to the Department of Pediatric and Preventive Dentistry, with a chief complaint of discoloration of teeth and poor esthetics due to irregular teeth in the upper and lower front region of jaw. There was no history of trauma in either the primary or permanent dentition. Family history did not reveal any evidence of hereditary dental anomalies. Clinical examination revealed multiple dental anomalies in both the maxillary and mandibular anterior region. Hypoplastic, irregular globular mass on labial surfaces of maxillary right central, lateral and canine (A). Similar irregular hypoplastic mass was seen on the cervical one-third of mandibular right central and lateral incisors (B). The presence of a talon cusp in the permanent maxillary right central incisor interfered with occlusion in the lower right incisor region (C). Dens invaginatus with maxillary permanent right lateral incisor was seen radiographically (D). Management of hypoplastic, irregular globular masses on labial surfaces of maxillary and mandibular teeth was done initially by grounding the tooth surfaces and reformed by composite build-up.

**Figure 1:**
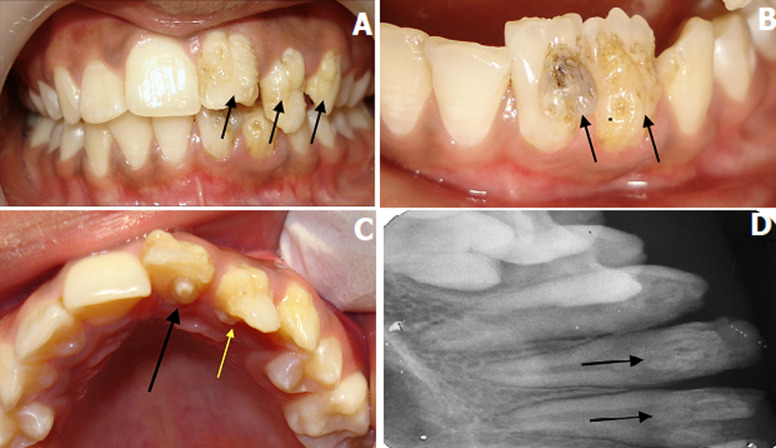
A) hypoplastic, irregular globular mass on labial surfaces of the maxillary right central, lateral, canine; B) crown dilacerations with permanent mandibular right central and lateral incisors along with labial hypoplastic mass; C) the black arrow denotes talon cusp in the permanent maxillary right central incisor, the yellow arrow denotes the dens invaginatus with the maxillary permanent right lateral incisor; D) intraoral periapical radiograph showing dens invaginatus in maxillary tooth

